# Selective and comprehensive analysis of organohalogen compounds by GC × GC–HRTofMS and MS/MS

**DOI:** 10.1007/s11356-015-5059-5

**Published:** 2015-07-22

**Authors:** Shunji Hashimoto, Yasuyuki Zushi, Yoshikatsu Takazawa, Teruyo Ieda, Akihiro Fushimi, Kiyoshi Tanabe, Yasuyuki Shibata

**Affiliations:** 1 National Institute for Environmental Studies, Onogawa 16-2, Tsukuba, 305-8506 Japan; 2 Advanced Industrial Science and Technology, Onogawa 16-1, Tsukuba, 305-8569 Japan

**Keywords:** Mass defect, Multidimensional data analysis, Neutral loss, Non-target analysis, Software extraction and cleanup

## Abstract

Thousands of organohalogen compounds, including hazardous chemicals such as polychlorinated biphenyls (PCBs) and other persistent organic pollutants (POPs), were selectively and simultaneously detected and identified with simple, or no, purification from environmental sample extracts by using several advanced methods. The methods used were software extraction from two-dimensional gas chromatography–high-resolution time-of-flight mass spectrometry (GC × GC–HRTofMS) data, measurement by negative chemical ionization with HRTofMS, and neutral loss scanning (NLS) with GC × GC–MS/MS. Global and selective detection of organochlorines and bromines in environmental samples such as sediments and fly ash was achieved by NLS using GC × GC–MS/MS (QQQ), with the expected losses of ^35^Cl and ^79^Br. We confirmed that negative chemical ionization was effective for sensitive and selective ionization of organohalogens, even using GC × GC–HRTofMS. The 2D total ion chromatograms obtained by using negative chemical ionization and selective extraction of organohalogens using original software from data measured by electron impact ionization were very similar; the software thus functioned well to extract organohalogens. Combining measurements made by using these different methods will help to detect organohalogens selectively and globally. However, to compare the data obtained by individual measurements, the retention times of the peaks on the 2D chromatograms need to match.

**Electronic supplementary material:**

The online version of this article (doi:10.1007/s11356-015-5059-5) contains supplementary material, which is available to authorized users.

## Introduction

We are surrounded by a variety of chemicals, and indeed, our lives are supported by huge numbers of man-made chemicals such as industrial chemicals, pharmaceuticals, and agrochemicals. However, some of these chemicals have caused, or are currently causing, environmental pollution or are having adverse effects on living organisms. Most persistent organic pollutants (POPs) including dichlorodiphenyltrichloroethane (DDT), hexachlorocyclohexane (HCH), and polychlorinated biphenyls (PCBs) were initially produced as useful and beneficial chemicals. Those pollutants suggest that some halogenated compounds have the potential for adverse effects on human and environmental health. Unfortunately, the types and concentrations of halogenated compounds in the environment are not fully known, because as yet, there is no measure that can detect all halogenated compounds and identify each of them.

Global detection, which can be used as a non-target analysis to search for large numbers of substances simultaneously, is one approach to addressing the increasingly diverse range of environmental pollutants. Direct measurement of samples without any loss of compounds is ideal for complete, global detection of pollutants. However, the conventional gas chromatograph, which is the mainstream tool for analyzing environmental pollutants, cannot separate the huge numbers of compounds contained in a crude sample. In recent years, comprehensive two-dimensional (2D) gas chromatography (GC × GC) has been used to characterize hundreds, or perhaps thousands, of petroleum chemicals (Blomberg et al. 1997; von Muhlen et al. 2006; Mao et al. 2009), as well as food components and flavors (Bicchi et al. 1999; Adahchour et al. 2002; Tranchida et al. 2010). GC × GC technology has also been used to analyze environmental contaminants with many congeners, such as PCBs (Hyotylainen et al. 2002; Korytar et al. 2002; Focant et al. 2003, 2004; Kristenson et al. 2005), polybrominated diphenyl ethers (PBDEs) (Focant et al. 2003; Korytar et al. 2005), and polyaromatic hydrocarbons (PAHs) (Hyotylainen et al. 2002; Kallio and Hyotylainen 2007; Ochiai et al. 2007; Fushimi et al. 2012), as well as polychlorinated dibenzo-*p*-dioxins and dibenzofurans (PCDDs/Fs) (Korytar et al. 2004; Danielsson et al. 2005; Shunji et al. 2008; de Vos et al. 2011). Although most of these studies have focused on the quantification of individual isomers, reports of non-target analysis by using GC × GC–MS have been increasing in recent years (Hilton 2007; Hilton et al. 2010; Pena-Abaurrea et al. 2014).

In our current studies, we are developing new apparatus consisting of GC × GC directly coupled with quadrupole-type tandem mass spectrometry (MS/MS; QQQ) or high-resolution time-of-flight mass spectrometry (HRTofMS), or both. Here, we present the results of one of our studies on the comprehensive and selective detection of halogenated compounds in environmental samples. The method is based on neutral loss scanning (NLS) with GC × GC–MS/MS and post-processing of data from GC × GC–HRTofMS with laboratory-built software. This technique has been reported in our previous papers (Hashimoto et al. 2011, 2013). Additionally, we report the selective detection of organohalogens by using negative chemical ionization (NCI) on GC × GC–HRTofMS and comparison of the results with selective data extracted by using the software. Electron-capture negative ionization, which is a function of NCI reactions, is an effective ionization method for electrophilic molecules such as organohalogens and nitro compounds.

## Materials and methods

### Chemicals

Carbon-13-labeled and carbon-13-unlabeled polychlorinated dibenzo-*p*-dioxins (PCDDs) were obtained from Wellington Laboratories Inc. (Guelph, ON, Canada); PCDFs, PCBs, and PBDEs from Cambridge Isotope Laboratories Inc. (Tewkesbury, MA, USA); other ^13^C-labeled and ^13^C-unlabeled POPs from Wako Pure Chemical Industries, Ltd. (Osaka, Japan); and 263 unlabeled pesticides from Kanto Chemical Co., Inc. (Tokyo, Japan).

### Samples

Certified reference materials provided by our institute were used for measurement as environmental samples. Nos. 17 and 20 were fly ash extract and sediment (note that these samples are currently not available because of low stocks). Soil samples were collected from an industrialized area, dried naturally, and screened with a 320-mesh sieve. Sediment and soil samples were Soxhlet-extracted for 16 h and then cleaned up with only a sulfuric acid-silica gel column. Indoor and outdoor air samples were collected into a Tenax-TA tube (Gerstel GmbH & Co. KG, Mülheim an der Ruhr, Germany) in our laboratory and on the rooftop of a building at our institute for a week. Samples of approximately 300 ml of urine were collected from each of two healthy adult males and combined. A portion (approximately 50 ml) of the combined sample was extracted with hexane.

### Measurement by GC × GC–QQQ with NLS

Measurement instruments and conditions are summarized in Table 1. Crude extract solutions of sediments, soils, and fly ash, and the contents of the Tenax-TA tubes that had adsorbed the indoor and outdoor air, were quantified with an Agilent 7890GC (Agilent Technologies, Santa Clara, CA, USA) with a Zoex KT-2006 GC × GC system (Zoex Corporation, Houston, TX, USA) coupled with an Agilent 7000 QQQ with NLS mode (Shunji et al. 2008).

**Table 1:** GC × GC–MS/MS (QQQ) and TD-GC × GC–HRTofMS conditions used to analyze environmental samples

TD^a^	
Thermal desorption	Gerstel TDU Transfer mode: fix; temp.: 340 °C; desorption mode: splitless; sample mode: sample remove from 40 °C holding for 1 min (delay 0.5+ initial 0.5) to 180 °C at rate 720 °C min^−1^ holding for 0 min to 340 °C at rate 50 °C min^−1^ holding for 5 min
Cryo-focusing	Gerstel CIS4 Heater mode: standard, cryo-cooling: enables from 0 °C holding for 0.2 min (equilibrium 0.1+ initial 0.1) to 300 °C at rate 12 °C min^−1^ holding for 3 min
GC × GC	
Instrument	Agilent 7890 GC or Agilent 6890 GC
GC × GC	Zoex KT2004 (in 6890GC) or Zoex KT2006 (in 7890GC)
1st column	GL Science InertCap 5MS/Sil (45 m length, 0.25 mm i.d., 0.1-μm film thickness)
2nd column	SGE BPX-50 (1 m length, 0.1 mm i.d., 0.1-μm film thickness)
Oven program	From 70 °C holding for 1 min to 180 °C at rate 50 °C min^−1^ holding for 0 min to 230 °C at rate 3 °C min^−1^ holding for 0 min to 300 °C at rate 5 °C min^−1^ holding for 16.133 min (total 50 min)
Injection	Volume: 1 μl, temp: 280 °C; method: splitless or solvent vent for TDU
Carrier gas	Type: He; mode: constant flow; initial head pressure: 246 kPa at 70 °C
Modulation	Period: 4 s; releasing: 0.25 s
MS/MS	
Instrument	Agilent 7000A QQQ (7000B equivalent)
Ion source	Mode: EI+; temp: 250 °C; ionizing voltage: 40 or 70 V; ionizing current: 35 μA
Analyzer	Mode: neutral loss scan, monitoring loss*: 19, 35, 37, 79, and 81 *m*/*z* Scan range: 150–530 *m*/*z*; cycle: 20 Hz
HRTofMS	
Instrument	JEOL JMS-T100GC or JEOL JMS-T100GCV 4G (NIES edition)
Ion source	i) mode: EI+; temp: 260 °C; ionizing voltage: 70 eV; ionizing current: 600 μA
	ii) mode: CI- (NCI); reaction gas; CH4 or Ar(trial); 0.5 ml/min; temp: 250 °C; ionizing voltage: 70 eV; ionizing current: 600 μA
	iii) mode: FI+; temp: 110 °C; counter electrode voltage: −10,000 V
Analyzer	Mass resolution: 8000–10,000 (best effort); recording range: 30–600 *m*/*z*; cycle: 33 Hz
Detector	MCP voltage: 2000–2400 V

^a^Thermal desorption (TD) was used for desorption of air samples

### Measurement by GC × GC–HRofMS

The same samples used for measurement by GC × GC–MS/MS, and the human urine samples, were newly quantified with an Agilent 6890GC with a Zoex KT-2004 GC × GC system coupled with a JEOL JMS-T100GC (JEOL Ltd., Tokyo, Japan), or with an Agilent 7890GC with a Zoex KT-2006 GC × GC system coupled with a JEOL JMS-T100GCV 4G (Hashimoto et al. 2011, 2013).

For measurement with the JMS-T100GCV 4G, we also employed NCI for selective ionization of organohalogens, as well as electron ionization (EI).

### Processing for extraction of organohalogens from GC × GC–HRTofMS data

We developed software that extracts only the mass spectra of organochlorines or organobromines from the data measured by the GC × GC–HRTofMS system. The software reads a netCDF file as input data, and extracts from the whole data set only those mass spectra that have chlorine or bromine isotopic patterns. For this process, the software never requires target mass setting (Hashimoto et al. 2013). It can vary the parameters—namely the mass accuracy, including mass resolution, mass range, threshold of signal intensity, and margin of error of the theoretical isotopic ratio of chlorine or bromine—for data extraction. It can also optionally pre-screen data by checking for mass defects, which are usually observed when the compound includes atoms such as halogens, and by using our method (Hashimoto et al. 2011), which can simulate NLS as a post-filter for the data.

In this software, mass deficiency is used for simple data filtration before Cl or Br isotopic pattern checking. Specifically, mass spectra with mass deficiencies within a range of 0 to −0.2 (the default setting for organochlorines) are left in the data, whereas the other spectra are removed. For example, the mass deficiencies of biphenyl (C_12_H_10_), pentachlorobiphenyl (PeCB, C_12_H_5_^35^Cl_5_), and pentabromobiphenyl (PeBP, C_12_H_5_^79^Br_5_) are +0.07825, −0.1166, and −0.3692, respectively; the mass deficiencies of H, ^35^Cl, and ^79^Br are +0.007825, −0.03118, and −0.08166, respectively. Therefore, application of a mass defect filter (MDF) with a range of 0 to −0.2 leaves the mass spectra of only PeCB; biphenyl and PeBP are removed from the data set. If the MDF is set to a range of 0 to −0.5, then, only biphenyl is removed from the data set. Incidentally, the mass deficiencies of four atoms of H and one atom of ^35^Cl, or of 10 atoms of H and one atom of ^79^Br, nearly balance.

## Results and discussion

### Selective detection of organohalogens by using NLS

**Fig. 1:**
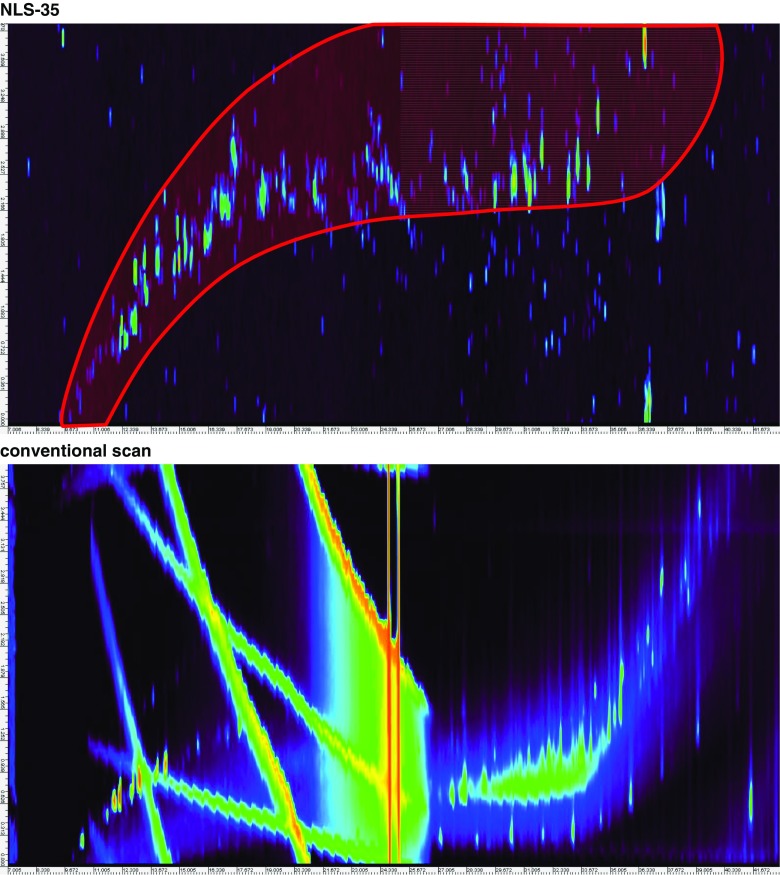
Two-dimensional total ion chromatograms (TICs) of a sediment sample (NIES CRM20), as measured by ^35^Cl neutral loss scanning (NLS-35, *upper*), which was expected to selectively detect organochlorines, and a conventional scan (*lower*) obtained by using GC × GC–MS/MS. The *red translucent shape* in the *upper* chromatogram shows the area where organohalogens were expected to appear

We successfully detected halogenated compounds comprehensively and selectively from environmental samples by using NLS with GC × GC–MS/MS (QQQ). The results of NLS for ^35^Cl (NLS-35), which was expected to selectively detect organochlorines by the loss of ^35^Cl from molecules, and a conventional scan (*m*/*z* = 150 to 530) of the sediment sample are shown as 2D total ion chromatograms (2D-TICs) in Fig. 1. Whereas a huge number of peaks and bands of complex compounds were observed in the chromatogram obtained with conventional scanning, many peaks were isolated by NLS-35 of the sediment sample. We then used NLS for ^79^Br (NLS-79), which we expected would selectively detect organobromines, as well as NLS-35, on a crude extract of fly ash. Successful global detection of halogenated compounds was demonstrated by NLS of these halogens using QQQ. However, the sensitivity of NLS is lower than that of conventional scan modes in general—it was 1/1000 to 1/100 as sensitive in the present case, depending on the conditions and the sample. This makes identification difficult, because proper mass spectra cannot be obtained from most peaks by using NLS. Alternatively, it was possible to search for, and identify, compounds by using 2D mass chromatograms and mass profiles obtained from measurements of the same sample with GC × GC–HRTofMS under the same conditions. In direct measurements performed on extracts from fly ash and sediment by using the above apparatus, many dioxin and PCB congeners were identified and many other halogenated compounds were found. A small number of chlorinated PAHs were also identified.

Precise retention-time matching of GC × GC peaks is important to enable the comparison of plural data and thus give better determination. A single gas chromatograph cannot separate the huge number of compounds contained in a crude sample, even when QQQ is used for detection. Therefore, the coupling of GC × GC and QQQ is an effective approach to complete, global detection of organohalogens.

### Selective extraction of organohalogens by using software

**Fig. 2:**
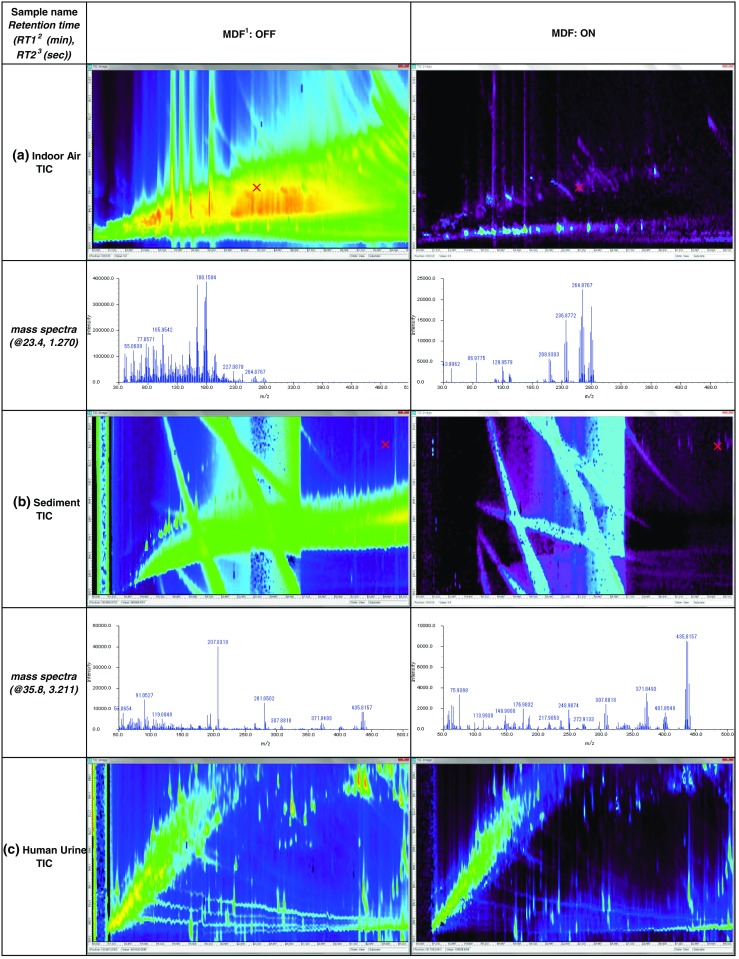
Comparison of the results of mass defect filter (*MDF*) pre-screening of data on three kinds of sample measured by GC × GC–HRTofMS. All total ion chromatograms were processed only by pre-screening using an MDF. Typical mass spectra were extracted by using our novel software under the same conditions, namely threshold 0; mass range, full; mass accuracy (MA), 0.05 u; extracted atom number, 3 to 10; and NLS, off, without MDF. ^1^MDF. Mass spectra with mass deficiencies within a 0 to −0.2 range were left in the data; other spectra were removed. ^2^Retention time (min) on the first gas chromatogram. ^3^Retention time (s) on the second gas chromatogram. *a* Indoor air TIC. *b* Sediment TIC. *c* Human urine TIC. This figure was reproduced from the work of Hashimoto et al. (2013)

We also developed a method that selectively extracts a subset of GC × GC–HRTofMS data to detect and identify trace levels of organohalogens (Hashimoto et al. 2013). By using our original software, namely the “Chlorine and Bromine isotopic profile Extractor” (CBEx), which finds typical peaks including mass clusters associated with the presence of chlorine isotopes, we achieved selective extraction of the mass spectra of organochlorines from the huge amounts of data obtained from environmental or biological samples by GC × GC–HRTofMS. Although Hilton (2007) have reported a similar data processing approach, they used unit mass data obtained by GC × GC–TofMS using a Leco Pegasus 4G. Moreover, Pena-Abaurrea et al. (2014) tried to find halogenated compounds in Ontario sediment samples by taking a scripting approach to Pegasus 4G data. To validate the effectiveness of using accurate mass for data extraction, we compared the results obtained by changing the mass width parameter of the software for data extraction. We found that high mass resolution and mass accuracy were valuable for selective extraction of organochlorines or organobromines. Screening by mass defect was effective for removing the mass spectra of hydrocarbons. Three applications of an MDF (one of the data-filtering functions of the software)—for the indoor air, sediment, and human urine data—are shown in Fig. 2. The mass spectra of an abundance of hydrocarbons and their fragments formed by EI were effectively removed by the MDF in the air and sediment samples (Fig. 2a, b). Comparison of the mass spectra before and after processing with the MDF clearly revealed the chlorine isotopic profile. The mass spectra of molecular sulfur in the sediment samples or in metabolites or biological derivatives in the human urine samples could not be removed by the MDF (Fig. 2b, c). Organochlorines were selectively and effectively extracted by the CBEx software after application of the MDF. Direct sample measurement and data extraction by using the software were therefore effective in non-target analysis.

### Selective ionization by NCI on GC × GC–HRTofMS

**Fig. 3:**
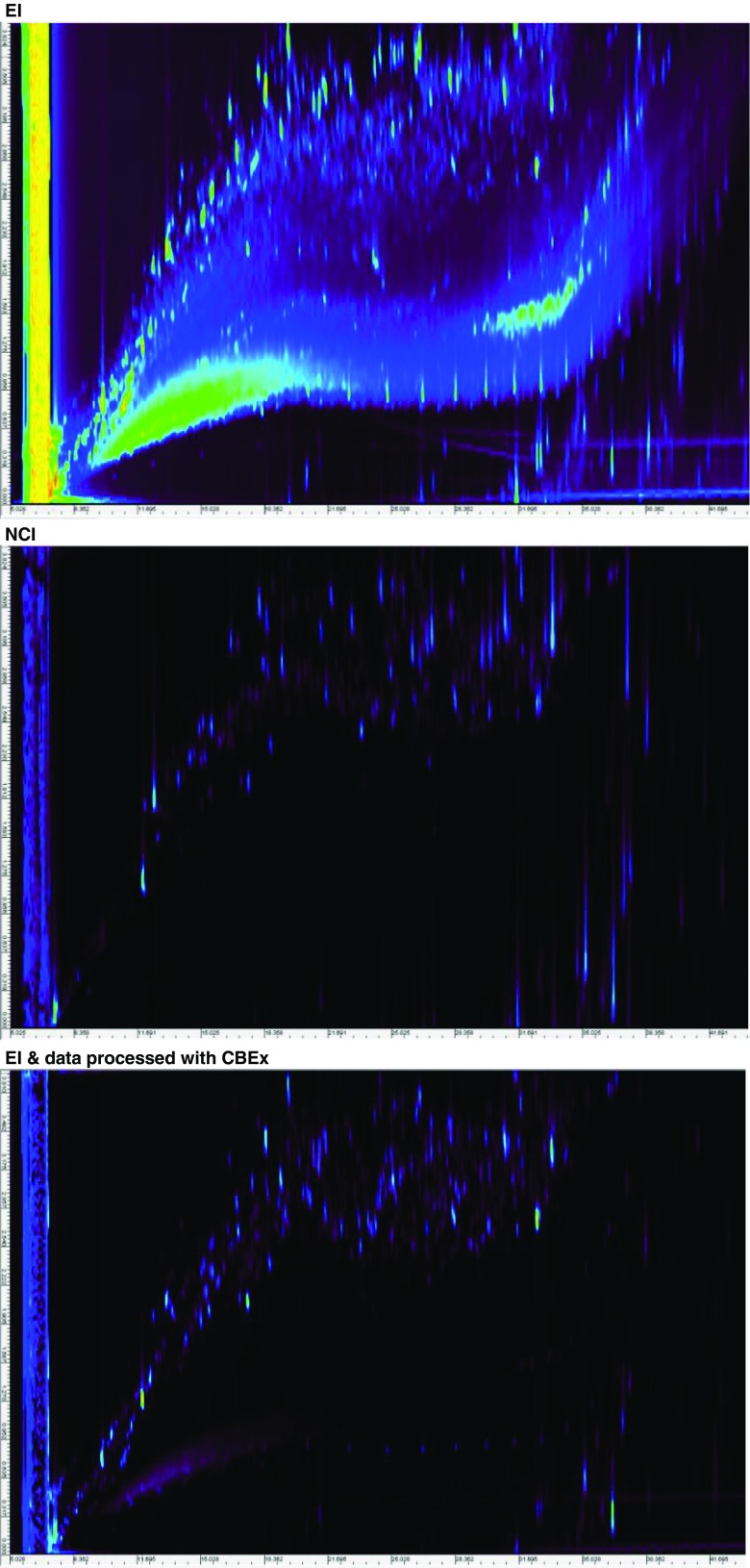
Two-dimensional total ion chromatograms (2D-TICs) of a soil sample, as measured by using electron ionization (*EI*), negative chemical ionization (*NCI*), and data measured with EI and processed for selective extraction of organohalogens by using our original software (*CBEx*). *Top*, 2D-TIC from EI; *middle*, 2D-TIC from NCI; *bottom*, 2D-TIC from processed EI data

**Fig. 4:**
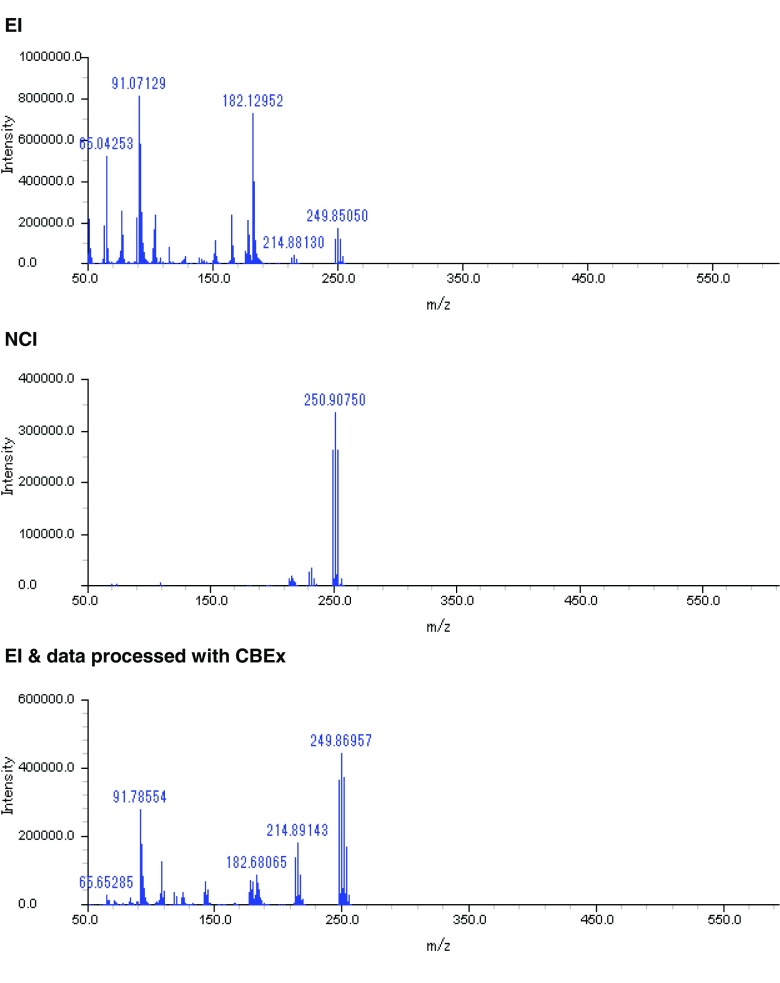
Mass spectra at the same location on 2D chromatograms of a soil sample, as measured by using electron ionization (*EI*), negative chemical ionization (*NCI*), and data measured with EI and processed for selective extraction of organohalogens by using original software (*CBEx*). *Top*, mass spectrum from EI; *middle*, mass spectrum from NCI; *bottom*, mass spectrum from processed EI data

We examined selective ionization by NCI as another approach to the global and selective detection of organohalogens, because it is known to be effective for ionization of these compounds (Cajka et al. 2005; Carrizo and Grimalt 2006; Hites 2008). The 2D total ion chromatograms of a soil sample measured with EI and NCI are shown in Fig. 3. Many peaks were observed in the EI chromatogram. Fewer peaks appeared in the middle 2D-TIC obtained by NCI; peaks that might have been derived from hydrocarbons and siloxanes were not observed. The bottom 2D-TIC shows the results of selective extraction of organohalogens by CBEx from the data measured with EI. When an MDF range of 0 to −0.2 was used, mass spectra that were likely representative of organochlorines were extracted. The middle and bottom TICs are very similar. Thus, both the software extraction and NCI were effective in detecting organohalogens selectively and comprehensively. The mass spectra of most of the peaks extracted by the software and detected by NCI were confirmed; these spectra have the typical cluster patterns of isotopes of chlorine or bromine. For example, the mass spectra of a peak located in the same position on each of the chromatograms are shown in Fig. 4. Cluster patterns of chlorine isotopes were observed in all the mass spectra. The NCI (middle) results show only a few mass spectra. A NIST 11 library search identified the processed spectrum (bottom) as pentachlorobenzene (MW = 250.3240), whereas the original spectrum (top) was regarded as bibenzyl (MW = 182.2660). This suggested that the mass spectra of pentachlorobenzene had been buried among the compounds in the EI analysis, even though it was measured by GC × GC. Even in this case, the mass spectra of molecular-related ions could be selectively obtained by NCI. Organohalogens can therefore be detected selectively and robustly by using NCI, but EI is important for identification by library searches. When enough separation cannot be achieved, even by using GC × GC, data processing after measurement helps to strip out the compound that has been specified as the target from the others. Table 2 shows the numbers of halogenated compounds estimated in the soil sample assessed by using EI (method 1 in the table) or NCI (method 3) and additionally extracted from those data by CBEx using only an MDF (methods 2 and 4) as an example. (Supporting data, including the results of a NIST library search, are listed in Appendixes A to D as supplementary information (SI).) Comparison of the numbers of organohalogens estimated by using methods 1 and 3 confirmed that NCI had greater organohalogen detection power than EI. The number of organohalogens estimated from the data extracted with CBEx (652 compounds were estimated as organohalogens by an NIST library search) was about twice that estimated from the original data (301 organohalogens estimated). This suggests that CBEx stripped out the mass spectra of the organohalogens from the co-eluted components, which were not separated—even by GC × GC. Table 3 lists the compounds estimated from a NIST library search of the data obtained by EI measurement and then processed or not processed with CBEx. A lot of organochlorines, including PCBs PCDDs, PCDFs, and chlorinated PAHs, were found from the data processed with CBEx; however, only a few organochlorines were estimated from the corresponding peaks in the unprocessed data. In contrast, we observed only a small difference in the number of organohalogens between the CBEx-extracted data and the original NCI data, suggesting that NCI is high selective.

**Table 2:** Numbers of compounds including the elements F, Cl, or Br in soil samples, as measured by using GC × GC–HRTofMS

Method	Number of organofluorine	Number of organochlorine	Number of organobromine	Total
1. EI^a^	103	137	61	301
2. EI > CBEx^c^	119	420	113	652
3. NCl^b^	71	302	100	473
4. NCl > CBEx	62	310	164	536

Compounds were estimated by a search of the NIST mass spectra library 2011 (NIST11)

^a^Sample was measured by using positive electron ionization

^b^Sample was measured by using negative chemical ionization

^c^Data were extracted by using only mass defect filtering (as part of our CBEx original software) after measurement by each type of ionization method

**Table 3:** List of the top 50 high-match compounds in a NIST library search

Peak ID	RT I^b^ (min)	RT II^c^ (s)	EI CBEx^a^		EI	
			Compound name	Match factor^d^	Compound name	Match factor
128	5.83	0.19	Ethylenediamine	762	Silane, dimethyl-	772
54	8.49	0.41	Benzene, tetrachloro-	772	Dichloro-1-oxa-2-sila-1,2-dihydronaphthalene	608
196	10.16	0.98	Naphthalene, dichloro-	767	1-Chloro-6-phenylhexane	576
232	10.56	1.14	Naphthalene, dichloro-	770	Fmoc-l-phenylalanine	584
231	11.49	1.17	Benzene, pentachloromethyl-	762	(na)^e^	
1	11.89	1.37	Benzene, hexachloro-	851	Benzene, hexachloro-	751
23	12.36	1.49	Naphthalene, trichloro-	793	Naphthalene, trichloro-	581
35	13.49	2.06	Naphthalene, trichloro-	725	Naphthalene, trichloro-	603
314	14.03	2.19	Pentachloroaniline	790	Acetic acid, cyclopropyl-(1,1′-biphenyl-4-yl)methyl ester	542
75	15.03	1.97	Naphthalene, tetrachloro-	777	Naphtho[2,3-b]norbornadiene	810
18	15.43	2.06	Naphthalene, tetrachloro-	880	Naphthalene, tetrachloro-	686
202	15.96	2.32	Naphthalene, tetrachloro-	877	Naphthalene, tetrachloro-	616
293	16.23	2.41	Naphthalene, tetrachloro-	809	Benzene, 1,1′-(2-methyl-1-propenylidene)bis-	595
183	16.63	2.60	Naphthalene, tetrachloro-	822	1,4,9(11)-Pregnatriene-3,20-dione, 21-acetoxy-17-hydroxy-	668
17	16.96	2.70	Naphthalene, tetrachloro-	849	Naphthalene, tetrachloro-	661
5	17.43	2.16	Benzene, pentachloro(trichloroethenyl)-	898	Benzene, pentachloro(trichloroethenyl)-	699
60	17.69	3.11	Naphthalene, tetrachloro-	846	Naphthalene, tetrachloro-	721
214	18.43	3.11	9H-Fluoren-9-one, dichloro-	745	2-Cyclohexen-1-one, 4,4-diphenyl-	604
987	18.69	1.24	Levoglucosenone	722	(na)	
821	18.89	1.17	1-(1-Methyl-2-piperidinyl)acetone	722	4,8,13-Cyclotetradecatriene-1,3-diol, 1,5,9-trimethyl-12-(1-methylethyl)-	772
1011	20.09	1.11	1-(1-Methyl-2-piperidinyl)acetone	741	(na)	
991	20.76	1.05	1-(1-Methyl-2-piperidinyl)acetone	773	2-Dodecen-1-yl(-)succinic anhydride	744
1050	22.03	1.02	(R)-1-Ethyl-2-pyrrolidinecarboxamide	740	2-Dodecen-1-yl(-)succinic anhydride	735
1111	22.16	1.02	3-(1′-Pyrrolidinyl)-2-butanone	741	Tetratriacontyl heptafluorobutyrate	779
1338	22.29	1.05	(R)-1-Ethyl-2-pyrrolidinecarboxamide	811	2-Dodecen-1-yl(-)succinic anhydride	738
950	22.49	1.02	1-(2-Tetrahydrofurylmethyl)piperidine	720	2-Dodecen-1-yl(-)succinic anhydride	737
298	22.89	2.54	Biphenyl, hexachloro-	812	Biphenyl, hexachloro-	589
1240	22.96	0.95	3-(1′-Pyrrolidinyl)-2-butanone	725	Tetrapentacontane, dibromo-	771
216	23.23	2.86	Tetrachlorodibenzofuran	828	2,6,10,14,18,22-Tetracosahexaene, 2,6,10,15,19,23-hexamethyl-, (all-E)-, didehydro deriv.	562
161	23.29	2.79	Tetrachlorodibenzofuran	817	Tetrachlorodibenzofuran	592
1283	23.36	0.98	3-(1′-Pyrrolidinyl)-2-butanone	766	Tricyclo[20.8.0.0(7,16)]triacontane, 1(22),7(16)-diepoxy-	735
47	23.76	2.79	Tetrachlorodibenzofuran	824	Tetrachlorodibenzofuran	644
238	24.36	3.97	1,2-Bis(2-chlorophenyl)-1,2-bis(3-chlorophenyl)ethane	725	11H-Benzo[a]fluoren-11-one	742
306	26.83	2.57	Biphenyl, heptachloro-	811	Biphenyl, heptachloro-	580
82	26.83	2.73	Pentachlorodibenzofuran	861	Pentachlorodibenzofuran	674
281	27.63	2.76	Pentachlorodibenzofuran	755	Molybdenum, dicarbonylbis(4-2-methylenecycloheptanone)-	546
382	28.23	2.57	Biphenyl, octachloro-	782	Agathic acid	522
389	28.43	2.60	Biphenyl, octachloro-	732	Biphenyl, octachloro-	513
279	29.49	2.54	Biphenyl, nonachloro-	806	Biphenyl, nonachloro-	605
207	29.76	2.67	Biphenyl, nonachloro-	815	Biphenyl, nonachloro-	620
358	30.36	2.70	Biphenyl, octachloro-	769	Biphenyl, octachloro-	582
122	31.56	2.73	Biphenyl, nonachloro-	881	Biphenyl, nonachloro-	665
2	32.63	2.83	Decachlorobiphenyl	752	Decachlorobiphenyl	739
4	33.36	3.43	Tetrachloro-1,3-disila-2-oxaphenalane	724	Tetrachloro-1,3-disila-2-oxaphenalane	610
29	35.09	0.70	Tetrachloro-1,3-disila-2-oxaphenalane	732	Tetrachloro-1,3-disila-2-oxaphenalane	564
194	36.36	3.87	Octachlorodibenzo-*p*-dioxin	814	Octachlorodibenzo-*p*-dioxin	635
3	36.56	0.41	Dibenzofuran, octachloro-	802	Dibenzofuran, octachloro-	776
1394	40.96	2.41	1H-Pyrazole, 4,5-dihydro-3-phenyl-	765	*O*-Phenylenspirobiindanol	525
677	42.63	2.70	9H-Xanthen-9-one, 2,7-dichloro-1-hydroxy-3,6-dimethoxy-8-methyl-	746	(na)	
393	44.09	4.03	1-(4-Methylpiperazine)dithiocarboxylic acid, 2,3,5,6-tetrachloropyrid-4-yl ester	797	(14β,20β,22R,25R)-3β-Hydroxy-5α-spirost-8-en-11-one	476

Soil sample data were processed with CBEx after measurement by EI mode; data were also extracted from the corresponding peaks before processing

^a^Data were extracted by using only mass defect filtering (as part of our original CBEx software) after measurement by EI

^b^Retention time (min) on the first gas chromatogram

^c^Retention time (s) on the second gas chromatogram

^d^Compounds were estimated by a library search with NIST 11 (NIST mass spectra library 2011)

^e^ *na* not applicable; corresponding peak was not found at the same retention times

Measurement of molecular ions is essential for identifying unknown compounds not registered in mass libraries. EI and NCI may not be enough; soft ionizations such as field ionization may be useful, despite insufficient ionization.

In conclusion, by using GC × GC combined with highly selective detection followed by sophisticated data analysis, we selectively and simultaneously detected and identified numerous organohalogen compounds, including hazardous chemicals such as PCBs and other POPs, without the need to purify environmental sample extracts. Software determination using GC × GC–HRTofMS data, analysis via NCI with HRTofMS, and NLS with GC × GC–MS/MS were shown to be effective methods. In addition, deconvolution of the peaks and mass spectra effectively improved data extraction performance, because many compounds were co-eluted even when GC × GC was used, depending on the sample matrix. Further development of the hardware to improve its accuracy and precision, together with enhancement of the software to improve the matching of retention times in 2D chromatograms to those in GC × GC–HRTofMS spectra, will make it possible to simultaneously detect and quantify even more compounds.

## Electronic supplementary material

Below is the link to the electronic supplementary material.

ESM 1 (XLSX 762 kb)
